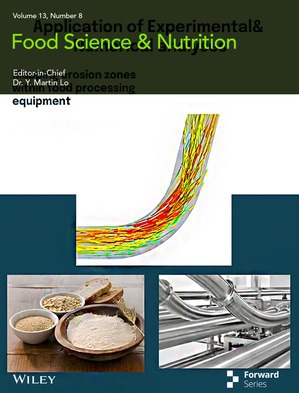# Cover Image

**DOI:** 10.1002/fsn3.70812

**Published:** 2025-11-27

**Authors:** Ali Ebadi, Adel Rezvanivand Fanaei, Ali Hassanpour, Vahid Rostampour

## Abstract

The cover image is based on the article *A Numerical‐Experimental Assessment of the Dilute Phase and Erosion in a Larvae‐Killing Processing System: Considering the Geometry Variation* by Ali Ebadi et al., https://doi.org/10.1002/fsn3.70771.